# Association between inflammatory cytokines and symptoms of major depressive disorder in adults

**DOI:** 10.3389/fimmu.2023.1110775

**Published:** 2023-02-13

**Authors:** Xue Min, Genwei Wang, Yalian Cui, Peipei Meng, Xiaodong Hu, Sha Liu, Yanfang Wang

**Affiliations:** ^1^Department of Microbiology and Immunology, School of Basic Medical Sciences, Shanxi Medical University, Taiyuan, China; ^2^Department of Psychiatry, First Hospital of Shanxi Medical University, Taiyuan, China; ^3^Shanxi Key Laboratory of Artificial Intelligence Assisted Diagnosis and Treatment for Mental Disorders, First Hospital of Shanxi Medical University, Taiyuan, China

**Keywords:** inflammatory cytokines, major depressive disorder, biomarkers, interleukins, immune

## Abstract

**Objective:**

This study investigated the association between inflammatory cytokines and major depressive disorder.

**Methods:**

Plasma biomarkers were measured by enzyme-linked immunosorbent assay (ELISA). Statistical analysis of baseline biomarkers in the major depression disorder (MDD) group and healthy controls (HC) group, and differences in biomarkers before and after treatment. Spearman analysis was performed to correlate baseline and after treatment MDD biomarkers with the 17-item Hamilton Depression Rating Scale (HAMD-17) total scores. Receiver operator characteristic (ROC) curves were analyzed for the effect of biomarkers on MDD and HC classification and diagnosis.

**Results:**

Tumor necrosis factor-α (TNF-α) and interleukin-6 (IL-6) levels were significantly higher in the MDD group than in the HC group, while high mobility group protein 1 (HMGB1) levels were significantly lower in the MDD group. The AUCs for HMGB1, TNF-α, and IL-6 were 0.375, 0.733, and 0.783, respectively, according to the ROC curves. MDD patients with brain-derived neurotrophic factor precursor (proBDNF) levels were positively correlated with total HAMD-17 scores. The levels of proBDNF levels were positively correlated with the total HAMD-17 score in male MDD patients, and brain-derived neurotrophic factor (BDNF) and interleukin 18 (IL-18) levels were negatively correlated with the total HAMD-17 score in female MDD patients.

**Conclusion:**

Inflammatory cytokines are associated with the severity of MDD, and TNF-α and IL-6 have the potential as objective biomarkers to aid in the diagnosis of MDD.

## Introduction

Major depressive disorder (MDD), or depression is a common and highly disabling mental disorder in which patients have a high risk of disability and a low quality of life (1). often occurring together with cardiovascular disease, diabetes, and autoimmune diseases (2, 3). MDD imposes a significant emotional and socioeconomic burden, and The World Health Organization estimates that 322 million people around the world from depression, which is about 4.4% of the global population (4).

MDD is highly heterogeneous in terms of clinical features and pathobiological alterations, which makes 1/3 of patients unresponsive or ineffective to conventional treatments (5). Furthermore, as the current diagnosis of MDD is based only on the symptom dimension, this makes the whole diagnostic process somewhat subjective and leads to a considerable risk of misdiagnosis and suboptimal treatment (6). Exploration of biomarkers as indicators of normal biological processes, pathogenic processes, or drug responses to therapeutic interventions may help identify homogeneous patients with MDD (7), such as those who may be involved in inflammatory phenotypes, so that individualized treatment plans can be developed for these patients to improve treatment rates.

The pathogenesis of MDD is extremely complex, and biomarker studies currently involve five biological systems, such as immunoinflammatory, neurotrophic, neurotransmitter, neuroendocrine and metabolic systems. The mechanism of immune inflammation has become a research hotspot in recent years, and since Ur (8) and others proposed the cytokine hypothesis, more and more studies have confirmed that MDD is accompanied by immune abnormalities (9). Early, Maes et al (10) found elevated inflammatory factors and CRP in patients with depressive disorders. Indoleamine 2, 3-dioxygenase (IDO), which decomposed tryptophan, can be activated by high levels of inflammatory factors. IDO’s metabolite quinolinic acid (QA) presents neuroexcitatory toxicity, leading to the reduction of a large number of markers of neuroplasticity, such as brain-derived neurotrophic factor (BDNF) levels, as well as affecting neurogenesis (11). This process may be associated with depression-like behavior in patients. In addition, inflammatory cytokines can stimulate the activation of the hypothalamic-pituitary-adrenal (HPA) axis and inhibit negative feedback loops, leading to hyperglucocorticoidemia. Elevated cortisol levels have been repeatedly shown to cause mood symptoms and are thought to be another potential link between inflammation and major depression (12).

An increasing number of studies have also found abnormalities in peripheral biomarkers in MDD both at baseline and after treatment (13, 14), such as cellular inflammatory factors and neurotropism. Also, antidepressants have been shown to reduce peripheral biomarkers such as IL-6, IL-10, and TNF-α (15), and anti-inflammatory drugs combined with antidepressants can reduce biomarkers such as CRP and TNF-α and improve depressive symptoms (16, 17). Khandaker et al. (18) suggested that elevated levels of inflammatory markers and others may be the etiology of MDD. Therefore, their exploration may not only add objective markers for clinicians to diagnose MDD but also obtain data about the effectiveness of treatment.

In this study, inflammatory factors, neurotrophic factors, and inflammatory proteins, such as TNF-α, IL-4, IL-6, IL-10, IL-18, IL-23, proBDNF, BDNF, hs-CRP, and HMGB1, were chosen as targets for investigation based on the association between immune inflammation, the nervous system, and MDD in this study (19). In order to better understand the diagnosis and treatment of MDD, it is important to find diagnostic biomarkers and therapeutic response biomarkers for MDD.

## Methods

### Participants

The 113 study cases were outpatients and inpatients admitted to the Department of Mental Health, First Hospital of Shanxi Medical University, from January 2019 to December 2021, of which 22 patients had longitudinal data after treatment. Inclusion criteria:1) aged 18-55 years old; 2) met the diagnostic criteria of “MDD (current episode)” in the Diagnostic and Statistical Manual of Mental Disorders (DSM-IV); 3) the 17-item Hamilton Depression Rating Scale (HAMD-17) ≧ 17 at the time of enrollment; 4) had normal understanding ability; 5) patient meets the diagnosis and is willing to receive antidepressant treatment; 6) patients with a first episode or relapse who have not taken medication in the last month. Exclusion criteria:1) severe physical illnesses that could interfere with the study treatment; 2) pre-existing serious organic brain disease, serious mental illness (schizophrenia, etc.); 3) pregnant or lactating women, or planned pregnancy; 4) no previous convulsion-free electroconvulsive therapy (MECT).

Forty-one healthy volunteers recruited from surrounding communities during the same period were selected as the control group. Inclusion and exclusion criteria:1) age 18-55 years old; 2) no blood relationship with the patient; 3) no history of serious physical illness; 4) no history of psychiatric disorders and or family history; 5) non-pregnant or lactating women. This study was approved by the Ethics Committee, and all study participants signed an informed consent form.

### Demographic data and clinical assessment

General information on enrolled subjects was collected using the self-administered observation of affective disorders scale. The HAMD-17 scale was used to evaluate the severity of MDD. Semi-structured interviews were conducted with the Mini-International Neuropsychiatric Interview (MINI) Chinese version.

### Treatment

Fluoxetine and Fluvoxamine, two members of the selective 5-hydroxytryptamine reuptake inhibitors (SSRIs) class of medications, were primarily utilized for a 6-week therapy period. Each medication’s lowest effective therapeutic dosage, average therapeutic dose, and maximum effective therapeutic dose were given after one, two, and four weeks, respectively. Subjects would be assessed for HAMD-17 scores at baseline and after 6 weeks of treatment by the same follow-up physician.

### Specimen collection, storage, and testing

5-6 ml of fasting venous blood was collected from study subjects at week 0 and week 6, blood samples were centrifuged at 3500 r/min for 10 min. The supernate was separated, extracted, and placed at -80°C for measurement. Marker assays included brain-derived neurotrophic factor precursor (proBDNF), brain-derived neurotrophic factor (BDNF), hypersensitive C-reactive protein (hs-CRP), high mobility group protein 1 (HMGB1), tumor necrosis factor-α (TNF-α), interleukin 4 (IL-4), interleukin 6 (IL-6), interleukin 10 (IL-10), interleukin 18 (IL-18), and interleukin 23(IL-23).

### Instruments and reagents

The equipment used for the assay was ELx808 ELISA, the thermostat was Sanyo’s MIR-262, and the enzyme-linked immunosorbent assay (ELISA) was used to detect the biomarkers in plasma. (1) Kits: BDNF, TNF-α, IL-4, IL-6, IL-10, IL-18, IL-23, and HMGB1 are provided by Cloud-clone corp Wuhan. proBDNF and hs-CRP are provided by Shanghai Jianglai biotechnology. (2) Intra-batch coefficient of variation: BDNF, TNF-α, IL-4, IL-6, IL-10, IL-18, IL-23, HMGB1<10%, proBDNF, hs-CRP <9%. (3) Coefficient of variation between batches: BDNF, HMGB1, TNF-α, IL-4, IL-6, IL-10, IL-18, IL-23<12%, proBDNF, hs-CRP <11%. (4) Detection sensitivity: 0.1ng/mL for proBDNF, 11.3pg/mL for BDNF, 0.1mg/L for hs-CRP, 28.3pg/mL for HMGB1, 6.5pg/mL for TNF-α, 5.9pg/mL for IL-4, 3.2pg/mL for IL-6, 2.3pg/mL for IL-10 The results showed that IL-18 was 5.9 pg/mL and IL-23 was 3.1 pg/mL.

### Statistical analysis

All data were statistically analyzed by SPSS26.0. Count data such as gender and marriage were tested by the χ2 test. The normal distribution of measurement data was tested by the Shapiro-Wilk test. The normal distribution was expressed as mean± SD (`x ± s) using the paired t-test and independent sample t-test was used. The non-normal distribution was represented as Median (IQR 25–75) employing the Wilcoxon signed-rank sum test or Mann-Whitney U test was used. Bivariate correlation analysis was performed using Spearman correlation. The ROC curve was employed to analyze the effect of biomarkers on the classification and diagnosis of MDD patients and HC. The scatter plot and ROC curve were plotted by GraphPad Prism 8.0. *p*<0.05 was considered as the level of statistically significant difference.

## Results

### Comparison of demographic data

There were no statistically significant differences in gender (χ2 = 1.678, *p*=0.194) and age (z=-0.127, *p*=0.227) between the MDD group and the HC group, while there were statistically significant differences in terms of marriage (χ2 = 16.134, *p*<0.001), education years (z=-2.298, *p*=0.022), and in HAMD-17(z=-9.439, *p*<0.001) (*p*<0.05) (**Table 1**).

**Table 1:** Comparison of demographic data between the MDD group and HC group.

Demographic variables	MDD (N=113) Median (IQR 25–75)	NC (N=41) Median (IQR 25–75)	*χ2/z*	*p*-value
Sex (male/female)	42/71	21/20	1.678	0.194
Marital state (married/unmarried)	90/23	19/22	16.134	0
Age (years)	27 (24,42)	31 (25,35)	-0.127	0.227
Education (years)	14 (12,15)	15 (14,15.5)	-2.298	0.022
HAMD-17	20 (18, 23)	0 (1, 2)	-9.439	0

MDD, major depressive disorder; HC, healthy control.

### Comparison of baseline biomarkers

**Figure 1:**
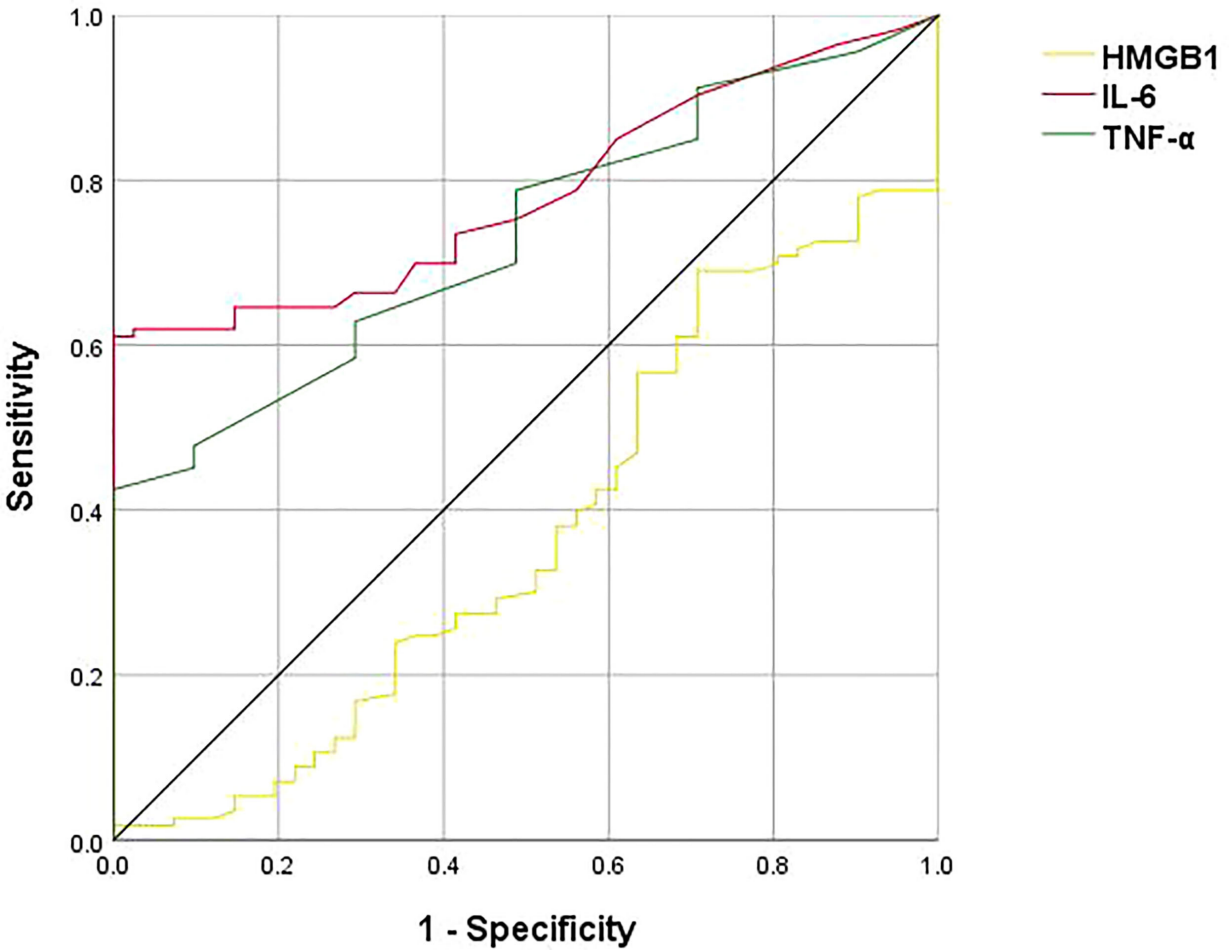
ROC curve analysis of HMGB1, TNF-α, IL-6, the black line represents HMGB1, the green line represents TNF-α, and the red line represents IL-6. The ROC curves showed that the AUCs of HMGB1, TNF-α, and IL-6 are 0.375, 0.733, and 0.783, respectively.

There were significant differences in plasma HMGB1 (t=-2.359, *p*=0.018), TNF-α (t=-4.431, *p*<0.001) and IL-6 (z=-5.372, *p*<0.001) levels between the MDD group and the HC group (**Table 2**). ROC curve results showed that the AUC of HMGB1, TNF-α and IL-6 were 0.375, 0.733 and 0.783, respectively (**Figure 1**).

**Table 2:** Comparison of biomarkers between the MDD group and HC group.

Project	MDD (N=113) Mean ± SD/Median (IQR 25–75)	HC (N=41) Mean ± SD/Median (IQR 25–75)	*p*-value
proBDNF (ng/mL)	2.936 ± 1.556	2.602 ± 1.330	0.368
HMGB1 (pg/mL)	117.995 ± 28.077	130.194 ± 26.379	0.018
TNF-α (pg/mL)	13.347 ± 1.241	12.901 ± 0.227	0
BDNF (pg/mL)	3.900 (2.491, 6.223)	3.19 (0.2.313, 4.784)	0.209
hs-CRP (mg/L)	2.347 (2.003, 2.656)	2.300 (1.500, 2.675)	0.463
IL-4 (pg/mL)	6.640 (4.494, 8.042)	6.640 (4.081, 9.097)	0.974
IL-6 (pg/mL)	2.106 (0.426, 2.727)	0.421 (0.391, 0.572)	0
IL-10 (pg/mL)	3.130 (2.699, 3.594)	3.189 (2.752, 3.956)	0.280
IL-18 (pg/mL)	3.465 (2.354, 5.690)	4.104 (2.196, 5.714)	0.792
IL-23 (pg/mL)	2.776 (2.286, 3.415)	2.570 (2.071, 3.195)	0.944

MDD, major depressive disorder; HC, healthy control; proBDNF, brain-derived neurotrophic factor precursor; HMGB1, high mobility group protein 1; TNF-α, tumor necrosis factor-α; BDNF, brain-derived neurotrophic factor; hs-CRP, hypersensitive C-reactive protein; IL, interleukin.

### Correlation between baseline biomarkers and HAMD-17

**Figure 2:**
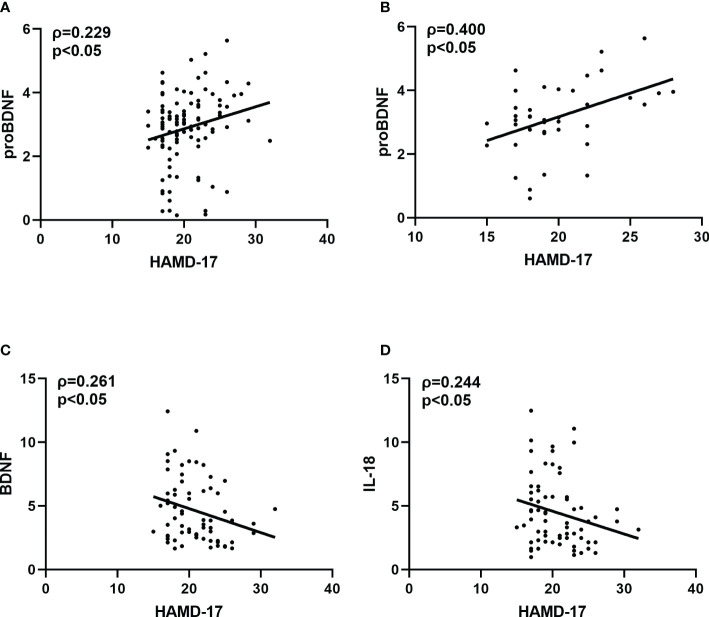
**(A)** Correlation between HAMD-17 and proBDNF in overall MDD patients. **(B)** Correlation between HAMD-17 and proBDNF in male MDD patients. **(C)** Correlation between HAMD-17 and BDNF in female MDD patients. **(D)** Correlation between HAMD-17 and IL-18 in female MDD patients. HAMD-17, 17-item Hamilton Depression Rating Scale proBDNF, brain-derived neurotrophic factor precursor; BDNF, brain-derived neurotrophic factor IL, interleukin.

Spearman correlation analysis showed a positive correlation between PROBDNF levels and total HAMD-17 score in all MDD patients (ρ=0.229, *p*<0.05). After gender grouping, there was a significant positive correlation between proBDNF and HAMD-17 scores in male MDD patients (ρ=0.400, *p*<0.05), a negative correlation between BDNF levels and HAMD-17 scores in female MDD patients (ρ=-0.261, *p*<0.05), and IL-18 levels and HAMD-18 scores in female MDD patients were negatively correlated (ρ=-0.244, *p*<0.05) (**Figure 2**).

### Comparison of HAMD-17 baseline and after treatment

**Figure 3:**
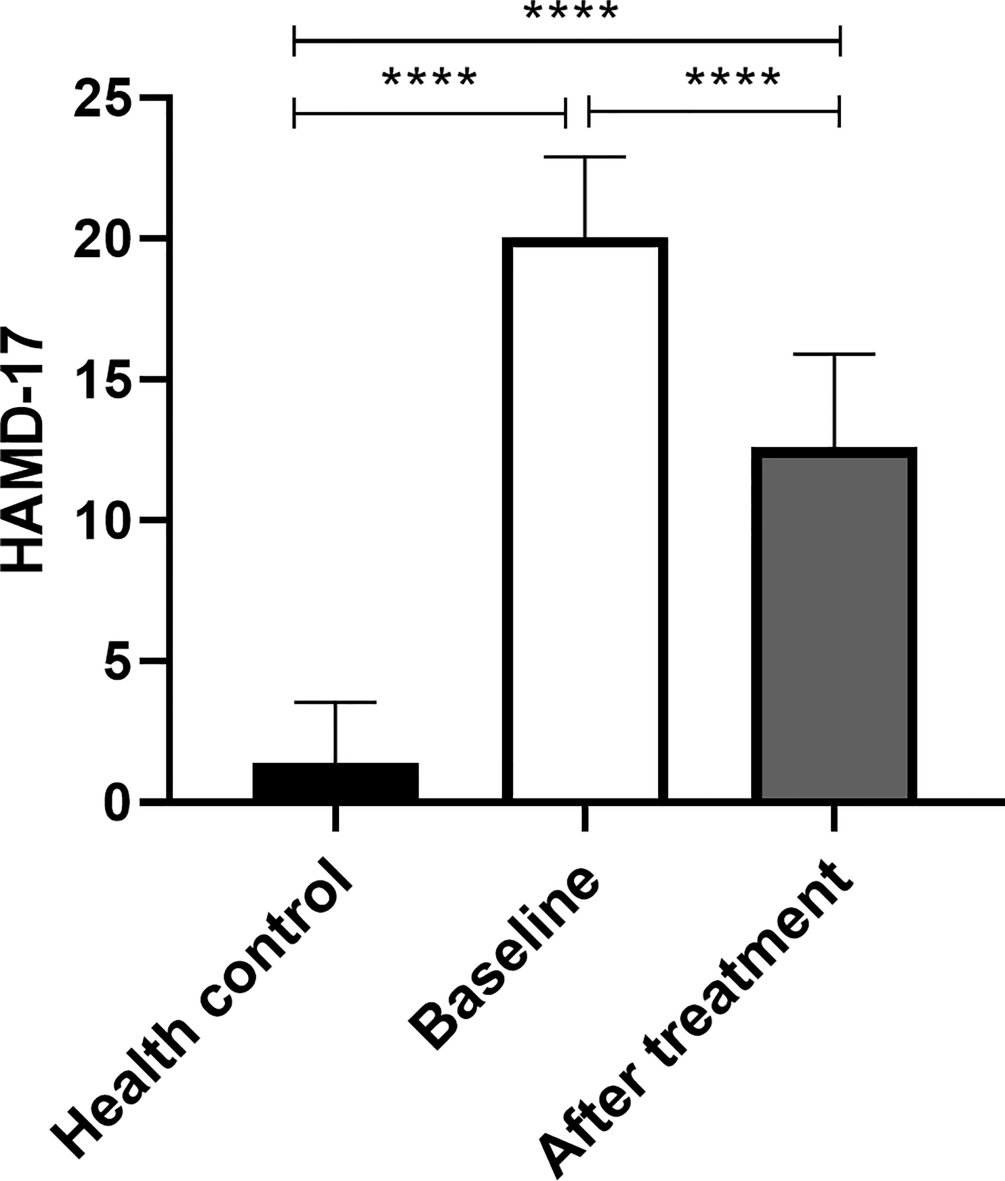
HAMD-17: 17-item Hamilton Depression Rating Scale. ****p<0.01.

HAMD-17 score at the baseline of MDD was higher than that at HC, and the difference was statistically significant (*p*<0.05). HAMD-17 score after MDD treatment was lower than baseline, the difference was statistically significant (*p*<0.05). HAMD-17 score after MDD treatment was higher than that of HC, and the difference was statistically significant (*p*<0.05) (**Figure 3**).

### Comparison of biomarkers baseline and after treatment

The levels of plasma TNF-α (t=4.580, *p*<0.001) and IL-6 (z=-2.996, *p*<0.001) in MDD patients baseline and after treatment were higher than those in healthy controls, with no significant difference in biomarkers between baseline and after treatment (**Table 3**).

**Table 3:** Comparison of biomarkers baseline and after MDD treatment.

Project	Baseline (N=22) Mean ± SD/Median (IQR 25–75)	After treatment (N=22) Mean ± SD/Median (IQR 25–75)	HC (N=41) Mean ± SD/Median (IQR 25–75)	*p*-value_1_	*p*-value_2_	*p*-value_3_
proBDNF (ng/mL)	3.018 ± 1.232	2.897 ± 0.856	2.602 ± 1.330	0.668	0.246	0.351
BDNF (pg/mL)	4.510 ± 2.690	4.947 ± 2.262	4.101 ± 2.428	0.592	0.542	0.182
hs-CRP (pg/mL)	2.340 ± 0.681	2.316 ± 0.671	1.941 ± 1.048	0.995	0.126	0.134
HMGB1 (pg/mL)	120.047 ± 28.679	129.190 ± 34.203	130.194 ± 26.379	0.342	0.163	0.897
TNF-α (pg/mL)	13.298 ± 0.464	13.403 ± 0.429	12.901 ± 0.227	0.330	0	0
IL-4 (pg/mL)	6.971 (5.083, 7.986)	7.316 (6.563, 9.545)	6.640 (4.081, 9.097)	0.100	0.874	0.081
IL-6 (pg/mL)	2.184 (0.411, 2.883)	2.494 (2.339, 2.883)	0.421 (0.391, 0.572)	0.260	0.003	0
IL-10 (pg/mL)	3.299 (2.929, 3.561)	3.160 (2.957, 3.561)	3.189 (2.752, 3.956)	0.711	0.823	0.896
IL-18 (pg/mL)	4.185 (2.829, 8.285)	3.968 (2.898, 5.958)	4.104 (2.196, 5.714)	0.638	0.345	0.644
IL-23 (pg/mL)	3.293 (2.724, 4.019)	2.776 (2.286, 3.573)	2.570 (2.071, 3.195)	0.113	0.055	0.664

p-value_1_, Statistical values for baseline comparison after the treatment; p-value_2_, Statistical values baseline compared with healthy controls; p-value_3_, Statistical values for after treatment compared with healthy controls.

HC, healthy control; proBDNF, brain-derived neurotrophic factor precursor; BDNF, brain-derived neurotrophic factor; hs-CRP, hypersensitive C-reactive protein; HMGB1, high mobility group protein 1; TNF-α, tumor necrosis factor-α; IL, interleukin.

## Discussion

The findings of this study showed that MDD and HC had distinct baseline biomarkers, depression severity was associated with different biomarkers in MDD by gender, and that the levels of biomarkers remained unchanged after treatment.

This study discovered statistically significant differences in the levels of HMGB1, TNF-α, and IL-6 in patients with MDD at baseline compared to healthy controls. HMGB1, a late-stage inflammatory factor, can interact with early inflammatory factors such as interleukin and tumor necrosis factor. In addition, it can be released by different cell types such as tissue macrophages, astrocytes, and neurons, acting on microglia Mac-1 to mediate chronic neuroinflammation, leading to progressive neurodegeneration. Some studies have found that HMGB1 is involved in the development of several cognitive-emotional disorders and neurological diseases (20). According to animal research, raising extracellular HMGB1 levels in the hippocampus promoted depressive-like behavior by regulating microglia activation (21). Wang et al. (22) first reported an increase in both central and peripheral HMGB1 protein levels in a chronic unpredictable mild stress (CUMS)-induced depressive behavior model, while the present study found lower plasma HMGB1 levels in the MDD group than in normal controls at baseline. The differences in the above results are considered to be related to the different selection of study subjects. On the other hand, HMGB1, when released extracellularly, can bind to the surface receptors of intrinsic immune cells and activate a series of intracellular inflammatory response pathways, causing increased synthesis and release of inflammatory factors (23). Therefore, we hypothesize that the large amount of HMGB1 in MDD in this study bound to immune cell surface receptors, which in turn caused a further elevation of TNF-α and IL-6 levels. It should be noted that HMGB1 levels in the 113 MDDs in this study were lower than those in HC at baseline, whereas the treated 22 MDD patients did not differ significantly from HC before and after treatment. This disparity may be attributed to an insufficient number of patients, since only 22 MDDs’ longitudinal data were followed in this research owing to the high incidence of patient shedding. More patients should be followed up to validate the results.

TNF-α is a multifunctional signaling molecule with antiviral and immunomodulatory effects. IL-6 is mainly secreted by monocytes-macrophages, produced by Th2, and has functions such as regulating immune responses. TNF-α and IL-6 induce the production of indoleamine 2,3 -dioxygenase (IDO), leading to a decrease in tryptophan and the production of tryptophan metabolites, which are associated with depression (24). Several studies have found that plasma TNF-α and IL-6 levels are higher in MDD patients than in normal controls (25, 26). This is consistent with the results of the present report. The ROC curve results of this study showed that TNF-α and IL-6 can be used to identify and differentiate MDD and HC with good diagnostic and classification effects. Cytokines may be involved in depression pathogenesis by regulating monoamine neurotransmitter metabolism and influencing neuroendocrine function (27), therefore, TNF-α, and IL-6 levels may serve as quantitative indicators of MDD patients in their early stages and as objective biomarkers for the disease

High levels of inflammatory factors may mediate the effects of inflammation on the brain and are associated with the autonomic nervous system (19), and high inflammation levels decrease the number of neuroplasticity markers, such as BDNF levels and neurogenesis (11). In this study, the levels of inflammatory factors TNF-α, and IL-6 were elevated in the MDD group, and BDNF levels were not statistically significant compared to healthy controls, which was consistent with those of Sagud (28). However, several studies have shown lower BDNF levels in MDD than in controls (29, 30). In addition, CRP, a typical inflammatory factor, and whose levels can reflect the level of inflammation in the body, was not found to be increased in the present study, which is inconsistent with the findings of Howren et al. (31). The difference in the results of the above studies was considered to be due to the greater heterogeneity of MDD, as well as related to the fact that this study did not control for potential factors such as BMI and smoking.

Extracellular proteases can convert proBDNF to mature BDNF, which has opposite biological effects through the neurotrophic factor receptor p75 (p75NTR) and complex kinase receptor B (TrkB), respectively. These receptors are crucial to the pathophysiology of mood disorders and the therapeutic mechanisms of antidepressants and mood stabilizers (32). The correlation analysis of this study found a positive correlation between proBDNF levels and HAMD-17 scores in MDD patients. In addition, the present study considered various responses based on gender differences, and correlation analysis showed that proBDNF levels were favorably connected with HAMD-17 scores in male MDD patients, but BDNF levels and IL-18 levels were inversely correlated with HAMD-17 scores in female MDD patients. Previous studies have shown that age and gender have a significant effect on plasma cytokine levels (33). This disparity was attributed to psychological and biological differences (34), as well as the prevalence of female patients with MDD being higher than male patients in many studies (35, 36). This could be related to estrogen’s effect on women’s immune responses. The intricacy of immunology, neuroinflammation, and MDD is highlighted by these results, which also imply that diverse inflammation indicators may be the most useful tool for patient categorization and that future research on MDD biomarkers should take gender into account.

In this study, depressive symptoms were reduced in MDD patients after treatment, but plasma TNF-α and IL-6 levels were not statistically different from baseline or HC, whereas plasma pro-inflammatory factors were reduced in MDD patients treated with conventional antidepressants in a large number of studies but were not significantly different from healthy controls (37). The variations in the aforementioned findings might be attributed to the heterogeneity of MDD or the various antidepressant medication classes. Serotonergic antidepressants are believed to decrease Th2-mediated immune responses, while norepinephrine antidepressants are considered to suppress Th1-mediated immunological responses, according to Martino et al. (38) The 5-hydroxytryptaminergic antidepressants that were mostly utilized in this investigation may not suppress TNF-α and IL-6 that is released by Th1 cells, which may partially account for the lack of substantial changes between baseline and treatment-induced TNF-α and IL-6 levels. Currently, the neurotransmitter functions of norepinephrine and dopamine are the main targets of antidepressant treatment. The above findings suggest that in the search for new therapeutic targets for MDD, the role of inflammation and the immune system in the pathogenesis of MDD is growing, and we should recognize the immune-inflammatory phenotype of MDD to develop the best treatment plan for patients.

This study uses plasma, which has certain advantages, such as not being affected by disturbances caused by coagulation and technical problems caused by fibrin. However, there are certain limitations. First, the statistical analysis did not include gender, age, and BMI as covariates between groups, and therefore may have some influence on the results. Second, longitudinal outcomes before and after treatment were compared with the same previous control group and no placebo control group. This is not methodologically optimal. Finally, as a cross-sectional study, it did not elucidate the causal relationship between MDD and biomarkers.

In conclusion, the findings suggest that proBDNF, BDNF, and IL-18 are linked to clinical symptoms of MDD, and that TNF-α and IL-6 have the potential to serve as objective biomarkers for the diagnosis of MDD. Further studies on the relationship between more biomarkers and MDD are expected in the future to develop new and tailored diagnostic and therapeutic strategies for MDD patients.

## Data availability statement

The raw data supporting the conclusions of this article will be made available by the authors, without undue reservation.

## Ethics statement

The studies involving human participants were reviewed and approved by Scientific research ethics review committee of Shanxi Medical University. The patients/participants provided their written informed consent to participate in this study. Written informed consent was obtained from the individual(s) for the publication of any potentially identifiable images or data included in this article.

## Author contributions

Conceptualization: YW, XM. Data curation: XM, YC, PM, SL. Formal analysis: XM, XH. Funding acquisition: YW. Investigation: XH, YW. Methodology: GW, XM. Supervision: YW, GW. Writing-original draft: XM. Writing-review & editing: YW, GW, SL. All authors contributed to the article and approved the submitted version.
